# Thanks to Editorial Board of *Dementia & Neuropsychology*

**DOI:** 10.1590/1980-5764-DN-2026-A001

**Published:** 2026-03-23

The Editors of *Dementia & Neuropsychology* thank the **Deputy Editors** (2), **Associate Editors** (44), **Social Media and Visual Abstract Editor** (1), **Consulting Board members** (48), and **Referees** (188), who altruistically and without remuneration dedicated their time to the development of the journal’s editorial activities from January 1, 2025, to December 31, 2025.

Thank you very much!

## DEPUTY EDITORS

Eliane Correa Miotto

Mônica Sanches Yassuda

## ASSOCIATE EDITORS

Adalberto Studart-Neto

Analuiza Camozzato

Antonio Lucio Teixeira

Artur Martins Novaes Coutinho

Breno José Alencar Pires Barbosa

Breno Satler de Oliveira Diniz

Ceres Eloah de Lucena Ferretti

Claudia Berlim de Mello

Claudia Kimie Suemoto

Cláudia Maia Memória

Cleusa Pinheiro Ferri

Déborah Oliveira

Douglas Mendes Nunes

Eliasz Engelhardt

Elisa de Paula França Resende

Florindo Stella

Jacy Bezerra Parmera

Jerusa Smid

Juliana Nery de Souza-Talarico

Karolina Gouveia César Freitas

Leandro Tavares Lucato

Leonardo Cruz de Souza

Leonardo Ferreira Caixeta

Leonel Tadao Takada

Lucas Porcello Schilling

Maíra Okada de Oliveira

Maira Tonidandel Barbosa

Marcela Lima Silagi de Siqueira

Marcia Cristina Nascimento Dourado

Marcia Maria Pires Camargo Novelli

Marcio Luiz Figueredo Balthazar

Maria Niures Pimentel dos Santos Matioli

Mario Amore Cecchini

Mauricio Arcos-Burgos

Mirna Lie Hosogi Senaha

Nilton Santos Custodio Capuñay

Raphael Machado de Castilhos

Renata Kochhann

Roberta Diehl-Rodriguez

Rubens Gisbert Cury

Thaís Bento Lima da Silva

Valeska Marinho Rodrigues

Vitor Tumas

Wyllians Jose Vendramini Borelli

## SOCIAL MEDIA AND VISUAL ABSTRACT EDITORS

Ricardo Alvim

## CONSULTIVE BOARD

Alexandre Castro-Caldas

André Luis Fernandes Palmini

Andrea Camaz Deslandes

Andrew J. Lees

Benito Pereira Damasceno

Cláudia da Costa Leite

Claudia Sellitto Porto

Dora Selma Fix Ventura

Facundo F. Manes

Francisco de Assis Carvalho do Vale

Giacomo Rizzolatti

Helenice Charchat-Fichman

Henrique Cerqueira Guimarães

Howard Chertkow

Jamary Oliveira Filho

Jerson Laks

João Carlos Barbosa Machado

John R. Hodges

John C. Morris

Jorge Neval Moll Neto

José Luiz Sá Calvalcanti

Lea Tenenholz Grinberg

Leila Maria Cardao Chimelli

M.-Marsel Mesulam

Márcia Lorena Fagundes Chaves

Marcia Radanovic

Maria Alice de Mattos Pimenta Parente

Maria Rita dos Santos e Passos Bueno

Mayana Zatz

Michael D. Geschwind

Moyses de Paula Rodrigues Chaves

Orestes Vicente Forlenza

Patricio Fuentes

Paulo Caramelli

Paulo Henrique Ferreira Bertolucci

Paulo Roberto de Brito Marques

Renato Anghinah

Ricardo F. Allegri

Ricardo de Oliveira Souza

Sandra Weintraub

Sérgio Luís Blay

Sergio Teixeira Ferreira

Tânia Marcourakis

Tales Alexandre Aversi-Ferreira

Thomas H. Bak

Vilma Regina Martins

Wilson Jacob Filho

Yves Joanette

## REVIEWERS

Abdessamad Elmourid

Adalberto Studart-Neto

Alberto Guerrero Velázquez

Alberto Justo

Alessandro Sousa

Aline Cristina Gratão

Allan Gustavo Bregola

Alyt Oppewal

Ana Júlia Bom Fim

Ana Luisa Rugani

Ana Luiza Nunes Cunha

Ana Ottaviani

Ana Paula Bertholo

André Palmeira

Andrei Bieger

Andreia Paiva

Andrew Miguel

Antonio Lucio Teixeira

Antônio Serafim

Beatriz Santana

Benito Damasceno

Breno Barbosa

Bruno Iepsen

Cammy Leung

Carla Gentile Matas

Carlos Pomilio

Carlos Tomaz

Carolina Delgado

Catherine Alexander

Celi Andrade

Ceres Ferretti

Cesar Castello Branco Lopes

César Minoru Toita Koga

Claudia Memória

Claudia Drummond

Conrado Borges

Cristiano Aguzzoli

Daniel Teixeira-dos-Santos

Danilo Silva

Debora Guerini

Diana Bacelar

Diana Monteiro Quirino

Diego de Castro dos Santos

Eduarda Naidel Barbosa

Eduardo Perrone

Einstein Camargos

Elaine Santana

Elisa de Paula Resende

Elisabetta Farina

Elizabeth Kelson

Ellen Soares Ishigaki

Ellison Cardoso

Emily Schworer

Emily Munn

Fabiana da Mata

Fabiola Canali Prado

Fabiola Macruz

Fabrício Feltrin

Federico Bellelli

Felipe Sudo

Fernando Freua

Fernando Lazzaretti

Filipi Leles da Costa Dias

Flávia Rolim

Florindo Stella

Francesca Mariani

Franciele Rohden

Gabriela Cipolli

Gilberto Alves

Guilherme Bueno

Hassan Mansour

Helena Fussiger

Henrique Ballalai-Ferraz

Henrique da Silva

Igor Vilela Brum

Isabel de Almeida

Isabel Cabrera

Isabel Guimaraes

Isabela Thaís Machado de Jesus

Jacy Parmera

Jerusa Smid

Jéssica Wingerter Teixeira Coelho

Joana Senger

João Pedro Ferrari-Souza

Joao Salgado

Joel Macoir

José Arimatéa de Oliveira Nery Neto

Jose Luiz de Brito Alves

José Wagner Leonel Tavares-Junior

Juan Carlos Carbajal Silva

Juliana de Souza-Talarico

Juliano Victor Luna

Júlio César Claudino dos Santos

Julio Cesar Vieira

Karen Socher

Karla Giacomin

Karolina Cesar Freitas

Leda Talib

Leonardo Caixeta

Leonel Takada

Liana Fernandez

Lorena Barbosa

Loreto Olavarria

Lucas Martins

Lucas Mourão

Luciana Cassimiro

Luciane Viola Ortega

Luís Felipe Miranda

Luis yu

Luziany Araujo

Maira Okada de Oliveira

Mara L. Cordeiro

Marcela Silagi de Siqueira

Marcelo Kenzo Takahashi

Marcio Luiz Balthazar

Márcio Medeiros

Marco Tulio Gualberto

Marcos Oliveira

Mari Nilva Maia da Silva

Maria Alice Baptista

Maria Isabel de Freitas D’Avila

Maria Niures Matioli

Maria Otaduy

Maria Paula Foss

Maritza Pintado Caipa

Mateus Antunes

Mateus Rozalem-Aranha

Mauricio Teixeira

Mawli Lawson

Míriam Soares

Mobin haghdel

Monica Yassuda

Mônica Melo

Mostafa Almasi-Dooghaee

Mounia Amane

Nadine Endaya

Nancy Huang

Natalie Pereira

Nilton Custodio Capuñay

Norberto Frota

Patricia Buchain

Patrícia Freitas

Paula Charlott Moritz

Paulo Bertolucci

Paulo Henrique Lazzaris Coelho

Paulo Verlaine Azevedo

Pawel Larionow

Pedro Barbosa

Rafael Carra

Ram Kumar Pandian

Ramiro Reckziegel

Raphael Castilhos

Raphael Spera

Raphael Tuma

Raquel Costa

Raul Arizaga

Renata Kochhann

Renata Leoni

Ricardo Alvim

Roberta Diehl Rodriguez

Rosa Salinas

Samir Magalhaes

Sara Onnivello

Sarah Elmi

Sarah Sales

Satiko Andrezza Ferreira Takano

Sheila Trentin

Silvia Bolognani

Silvia Merlin

Silvia Rodrigo-Herrero

Simone Barreto

Stefano Cappa

Tamine Capato

Tatiana Santos

Tatiane da Silva

Thais Bento Lima da Silva

Thais de Faria

Tomas Leon

Valeria Serrao

Valeska Marinho Rodrigues

Victor Caixeta

Victor Calil

Victor Campos

Victor Dornelas

Vitor Tumas

Wendell Rabelo

William Flavin

Wyllians Borelli

