# Erratum: Thanks to Editorial Board of *Dementia & Neuropsychology*

**DOI:** 10.1590/1980-5764-DN-2026-A001-ER

**Published:** 2026-06-05

ERRATUM: Thanks to Editorial Board of *Dementia & Neuropsychology.* The manuscript "Thanks to Editorial Board of *Dementia & Neuropsychology*", DOI: https://doi.org/10.1590/1980-5764-DN-2026-A001, published in Dement Neuropsychol 2026;20:e2026A001.

**Where it reads:**

https://doi.org/10.1590/1980-5764-DN-2025-A001

e2025A001

**It should read:**

https://doi.org/10.1590/1980-5764-DN-2026-A001

e2026A001

Editor-in-Chief: Dra Sonia M. D. Brucki https://orcid.org/0000-0002-8303-6732

